# Loss of form vision impairs spatial imagery

**DOI:** 10.3389/fnhum.2014.00159

**Published:** 2014-03-19

**Authors:** Valeria Occelli, Jonathan B. Lin, Simon Lacey, K. Sathian

**Affiliations:** ^1^Department of Neurology, Emory UniversityAtlanta, GA, USA; ^2^Rehabilitation Medicine, Emory UniversityAtlanta, GA, USA; ^3^Psychology, Emory UniversityAtlanta, GA, USA; ^4^Rehabilitation R&D Center of Excellence, Atlanta VAMCDecatur, GA, USA

**Keywords:** imagery, blindness, sense of direction, cognitive style, spatial

## Abstract

Previous studies have reported inconsistent results when comparing spatial imagery performance in the blind and the sighted, with some, but not all, studies demonstrating deficits in the blind. Here, we investigated the effect of visual status and individual preferences (“cognitive style”) on performance of a spatial imagery task. Participants with blindness resulting in the loss of form vision at or after age 6, and age- and gender-matched sighted participants, performed a spatial imagery task requiring memorization of a 4 × 4 lettered matrix and subsequent mental construction of shapes within the matrix from four-letter auditory cues. They also completed the Santa Barbara Sense of Direction Scale (SBSoDS) and a self-evaluation of cognitive style. The sighted participants also completed the Object-Spatial Imagery and Verbal Questionnaire (OSIVQ). Visual status affected performance on the spatial imagery task: the blind performed significantly worse than the sighted, independently of the age at which form vision was completely lost. Visual status did not affect the distribution of preferences based on self-reported cognitive style. Across all participants, self-reported verbalizer scores were significantly negatively correlated with accuracy on the spatial imagery task. There was a positive correlation between the SBSoDS score and accuracy on the spatial imagery task, across all participants, indicating that a better sense of direction is related to a more proficient spatial representation and that the imagery task indexes ecologically relevant spatial abilities. Moreover, the older the participants were, the worse their performance was, indicating a detrimental effect of age on spatial imagery performance. Thus, spatial skills represent an important target for rehabilitative approaches to visual impairment, and individual differences, which can modulate performance, should be taken into account in such approaches.

## Introduction

Previous studies comparing spatial imagery abilities in the sighted and the blind have yielded inconsistent results (see Renzi et al., 2013 for a review), some showing little difference (Vanlierde and Wanet-Defalque, 2004; Vecchi et al., 2004; Giudice et al., 2011) but others reporting that the blind are impaired compared to the sighted (Vecchi, 1998; Aleman et al., 2001; Knauff and May, 2006; Cornoldi et al., 2009). For example, Vecchi (1998) found that the congenitally blind were less accurate than the sighted when tested on a task that involved learning target squares and pathways on haptic matrices. On a similar task, the congenitally blind were less accurate than the sighted when using a spatial imagery strategy but were comparable to the sighted when using a verbal strategy (Cornoldi et al., 2009). By contrast, blind and sighted participants showed equivalent performance on spatial judgments from different imagined perspectives on visually and haptically learned maps, and both groups exhibited evidence for spatial updating (Giudice et al., 2011). Vanlierde and Wanet-Defalque (2004) required early-, late-blind and sighted participants to imagine shapes in a matrix, following line-by-line descriptions as to whether each square was black or white, and to make symmetry judgments. Despite the demanding nature of the encoding process, the three groups performed similarly but employed different strategies, the late-blind and sighted using mental imagery while the early-blind used a coordinate system that was not visually based.

Experimental evidence indeed suggests that individual differences in preferences for object or spatial imagery could modulate performance on spatial tasks. Object imagers tend to produce pictorial images that are detailed, vivid, and include information about surface properties such as color and texture; in contrast, spatial imagers tend to generate more schematic images that focus on spatial relations between component parts and on spatial transformations (Kozhevnikov et al., 2005). Further, people also vary in their reliance on verbal coding. Unlike visualizers (who might tend to prefer either an object imagery or spatial imagery strategy), verbalizers rely primarily on verbal-analytical strategies (Kozhevnikov et al., 2002, 2005; Blazhenkova et al., 2006). The Object-Spatial Imagery and Verbal Questionnaire (OSIVQ) assesses individual preferences for particular cognitive styles: verbalizing, object imagery and spatial imagery (Blazhenkova and Kozhevnikov, 2009).

Although the object-spatial imagery continuum was originally described in relation to visual imagery, similar differences arise in haptically derived images and multisensory representations (Lacey et al., 2010, 2011), raising the possibility that individual differences may also modulate performance in the visually impaired. The results obtained in previous studies (Vanlierde and Wanet-Defalque, 2004; Cornoldi et al., 2009) seem to suggest that dissimilarities in the strategies employed may indeed mediate the observed performance differences between blind and sighted participants. For example, verbal strategies appear to be more effective than spatial strategies for early- and congenitally blind, whereas the opposite holds for the late-blind and the sighted (Vanlierde and Wanet-Defalque, 2004; Cornoldi et al., 2009). Despite the existence of previous research investigating the behavioral effects of reliance on different cognitive styles, the effect of individual preferences for object imagery, spatial imagery and verbalizing has not been investigated in the blind, to the best of our knowledge. Such preferences might relate to inter-individual variability in mobility skills (Loomis et al., 1993; Schmidt et al., 2013). The capability of people to acquire spatial knowledge and update their own location in space as a result of self-motion can be assessed by the Santa Barbara Sense of Direction Scale (SBSoDS; Hegarty et al., 2002). Since one's sense of direction derives from direct experience with the environment, correlation of SBSoDS scores with performance on a spatial task (e.g., Kozhevnikov et al., 2007; Palermo et al., 2008) is considered evidence of the ecological validity of the task.

Here, we examined how performance on a spatial imagery task, similar to those used in earlier studies, is affected by visual status or individual cognitive style, and how spatial imagery performance relates to a participant's sense of direction, by comparing a group of late-blind participants to an age- and gender-matched control group. We predicted that the blind would perform worse than the sighted overall, as in some previous studies pointing to the behavioral relevance of visual experience in the development of spatial imagery (Vecchi, 1998; Noordzij et al., 2007; Gandhi et al., 2014). This possibility is favored by the demonstrations that the blind tend to rely more on verbal strategies while performing this type of task, and that such strategies are detrimental for the construction of spatial representations (Cornoldi et al., 2009; Schmidt et al., 2013). Performance on this task is also expected to be modulated by imagery preference, with object imagers performing worse than spatial imagers, given the higher proficiency of the latter group at processing the spatial relationships between object parts (Kozhevnikov et al., 2005). Moreover, preferential reliance on verbal cognitive style is predicted to impair spatial imagery performance, consistent with previous evidence on the effect of cognitive style on map learning (Pazzaglia and Moè, 2013). Further, we expected that the SBSoDS score would correlate positively with spatial imagery performance, indicating ecological validity for our spatial imagery task (see Wolbers and Hegarty, 2010, for a review).

## Materials and methods

### Participants

Twenty blind participants 11 female; mean age 45 years, 5 months (*SD* 12 years) and 20 age- and gender-matched sighted controls 11 female; mean age 44 years, 8 months (*SD* 12 years) took part and were compensated for their time. The blind participants were recruited through advertisements placed with the Center for the Visually Impaired in Atlanta, Georgia, and the Georgia chapter of the National Federation of the Blind. Clinical and demographic details are provided in Table 1, which shows that the age at which the blind participants completely lost form vision ranged from 6 to 50. As is common in studies of the blind, many of them had some visual loss at birth that subsequently progressed for various reasons. At the time of the testing, none of them had form perception. The sighted controls were recruited through advertisements posted on the Emory campus and intranet. All participants gave informed written consent. For the blind group, either Braille versions of the consent documents were provided or the experimenter read the forms aloud to the participant prior to signature. All procedures conformed to the Declaration of Helsinki and were approved by the Emory University Institutional Review Board.

**Table 1:** **Demographic and clinical data for the blind participants**.

**Gender**	**Age**	**Etiology**	**Age at which form vision lost**
M	48	Choroideremia from birth	37
F	43	Glaucoma	10
M	39	Cataracts (4), detached retina (6)	6
M	26	Macular degeneration	9
F	41	Proliferative diabetic retinopathy and neurovascular glaucoma	30
M	55	Glaucoma (gradual)	16
F	62	Glaucoma, discovered during treatment for trauma (staple in eye)	27
M	50	Retinitis pigmentosa	37
F	60	Diabetic retinopathy, glaucoma	50
M	44	Glaucoma following cataract surgery trauma	40
F	29	Glaucoma	21
F	45	Retinopathy of prematurity, macular degeneration	21
M	34	Prematurity, glaucoma, cataracts, detached retina	31
M	45	Retinitis pigmentosa	31
F	37	Optic atrophy	21
F	59	Optic atrophy	7
F	50	Glaucoma at birth, trauma (hit with ball at around age 10)	10
F	22	Glaucoma	11
F	65	Glaucoma	50
M	54	Glaucoma	14

### Procedure

#### Questionnaire and self-report

Participants were scored on the SBSoDS, which uses a set of questions to assess real-world spatial navigation abilities (Hegarty et al., 2002). Sighted participants completed the OSIVQ (Blazhenkova and Kozhevnikov, 2009), so that their preferences for object imagery, spatial imagery and verbalizing could be assessed. The OSIVQ is a 45-item self-report instrument consisting of three scales, each composed of 15 items, assessing, respectively, object visualization style (e.g., “My images are very colorful and bright”), spatial visualization style (e.g., “My images are more like schematic representations”), and verbal style (e.g., “My verbal skills are excellent”). Participants were asked to read and rate each item of the questionnaire on a 5-point scale, with 1 = totally disagree and 5 = totally agree, and ratings 2–4 indicating intermediate degrees of agreement or disagreement. The total score on each of the three scales corresponds to the mean of the item scores for that scale. For each participant, the spatial imagery score was subtracted from the object imagery score such that a negative difference score (O-S) represents a preference for spatial imagery whereas a positive O-S represents a preference for object imagery, as described earlier (Lacey et al., 2011). Since the object-spatial dimension is a continuum rather than being simply dichotomous (Kozhevnikov et al., 2010), the difference score represents the relative weight of one type of imagery over another. In the absence of a standardized tool to assess preferential imagery strategies in blind people, we followed the three-dimensional model proposed by Blazhenkova and Kozhevnikov (2009) in order to build a cognitive style self-evaluation report. All participants read or were read a script (see Supplementary Materials), and were then asked to classify themselves as an object imager, a spatial imager or a verbalizer by assigning a proportion score (from 0 to 1 for each dimension, so that the total equaled 1) to each cognitive style. O-S scores for each participant were calculated from these scores as described above.

#### Spatial imagery task

**Figure 1:**
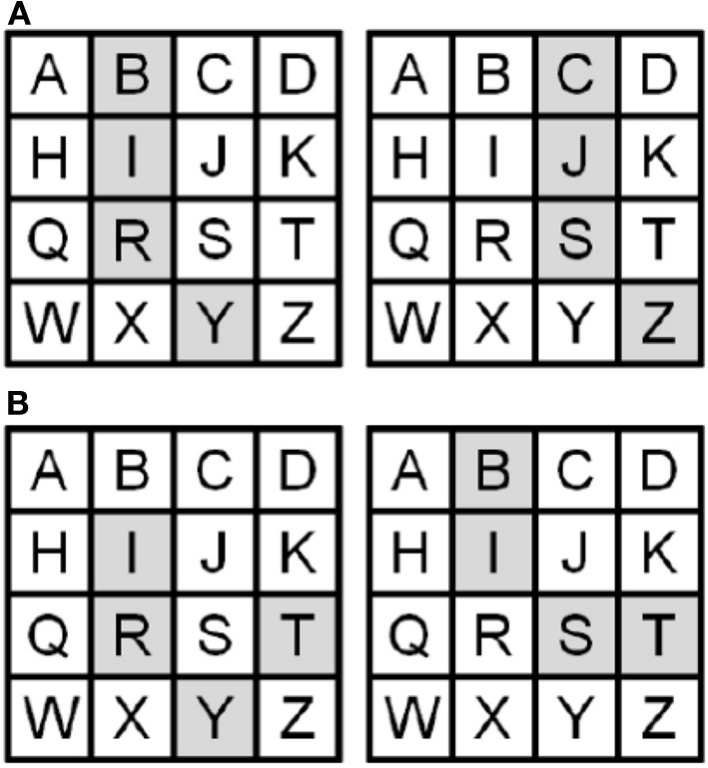
**Representative shapes in the lettered matrix with sample pairs of (A) “same” (B-I-R-Y vs. C-J-S-Z) and (B) “different” shapes (I-R-Y-T vs. B-I-S-T)**.

The spatial imagery task required imagining a previously memorized 4 × 4 matrix with one letter in each position (Figure 1). Participants imagined the shape that would result if four cells in the matrix, cued by auditory four-letter strings, were filled in, and performed a one-back same/different discrimination on the imagined shapes. Thus, participants had to compute global shape by processing the spatial relationships between component parts. To train participants on this task, we first asked them to memorize the lettered matrix. The sighted did this visually while the blind used a Braille version. For blind participants, the experimenter also read the letter aloud and guided the participant's hands on the Braille matrix to provide the relative spatial position of each letter; this was necessary for blind participants who could not read Braille and served as an additional aid for those who could. No time limit was set for this and no instructions were provided regarding the method of memorization, to allow for spontaneous use of individually preferred strategies. To test for accurate memorization, participants were asked to identify the four-letter sequences that formed all the horizontal rows, vertical columns, 2 × 2 squares, and diagonal lines. They then had to describe the shapes represented by the four-letter sequences, read aloud by the experimenter, for all the horizontal rows, vertical columns, and the 2 × 2 squares in the four corners of the grid (note that none of these shapes appeared in the main task). If errors were made, participants were allowed more time to study the matrix. When all these questions could be answered correctly, participants proceeded to the main task. This training procedure helped ensure similar encoding of the matrix in both sighted and blind participants despite the different modalities of presentation. Finally, participants were given a practice run of the actual task, as described below, to accustom them to generating images cued by four-letter strings at the speed of the actual experiment and without feedback about errors. For the actual spatial imagery task, participants completed 4 runs consisting of 6 blocks, each containing 3 trials (72 trials in all). In each block, they heard 4 four-letter strings, each lasting 4 s, with 3 s between each to respond by saying “same” or “different” relative to the immediately preceding stimulus. Thus, within each block, the second four-letter string was compared with the first, the third with the second and the fourth with the third. The one-back comparisons therefore required three responses in each 28 s block. There was a 15 s rest period between each block and each task run therefore lasted 243 s. On “same” trials, successive shapes were represented by differing letter sequences (Figure 1A), thus ensuring that participants had to construct mental images of the shapes and could not perform the task merely by comparing the letter strings. Since changing the letters necessarily changed the location of the shape in the matrix (Figure 1A), participants were instructed that they should make their decisions based on the shapes they constructed, ignoring their locations in the matrix. “Different” shapes were, of course, necessarily represented by different letters (Figure 1B). None of the letter strings resulted in a real word, thus restricting the possibility of verbal strategies, although some were pronounceable non-words, e.g., “B-I-S-T” (Figure 1B). The experiment was conducted using Presentation software (Neurobehavioral Systems Inc., Albany, CA).

## Results

**Figure 2:**
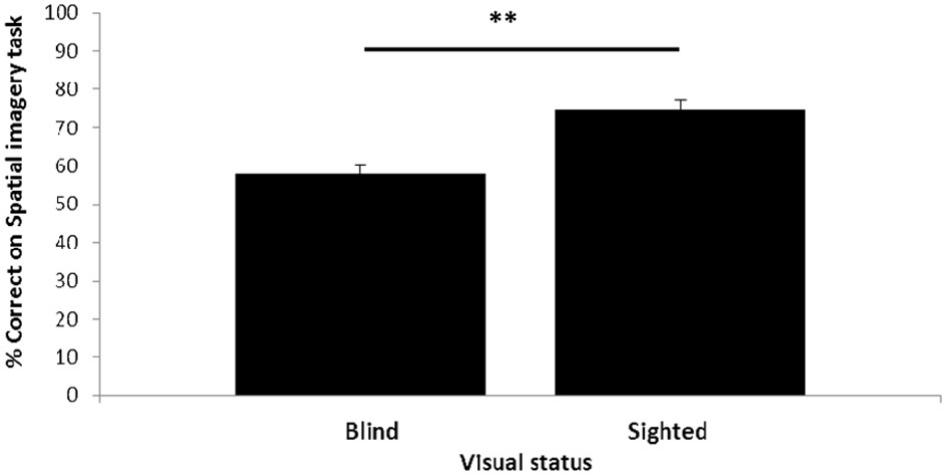
**Relationship between visual status and spatial imagery task performance**. Error bars: standard error of the mean (s.e.m.) ^**^p < 0.1.

Visual status had an impact on performance of the spatial imagery task (Figure 2). The blind participants, although performing significantly above chance [*t*_(19)_ = 3.40, *p* = 0.003], had lower accuracy (58%) than the sighted (74%); this difference was highly significant [*t*-test; *t*_(38)_ = −4.64, *p* < 0.001: all *t*-tests reported are two-tailed].

**Figure 3:**
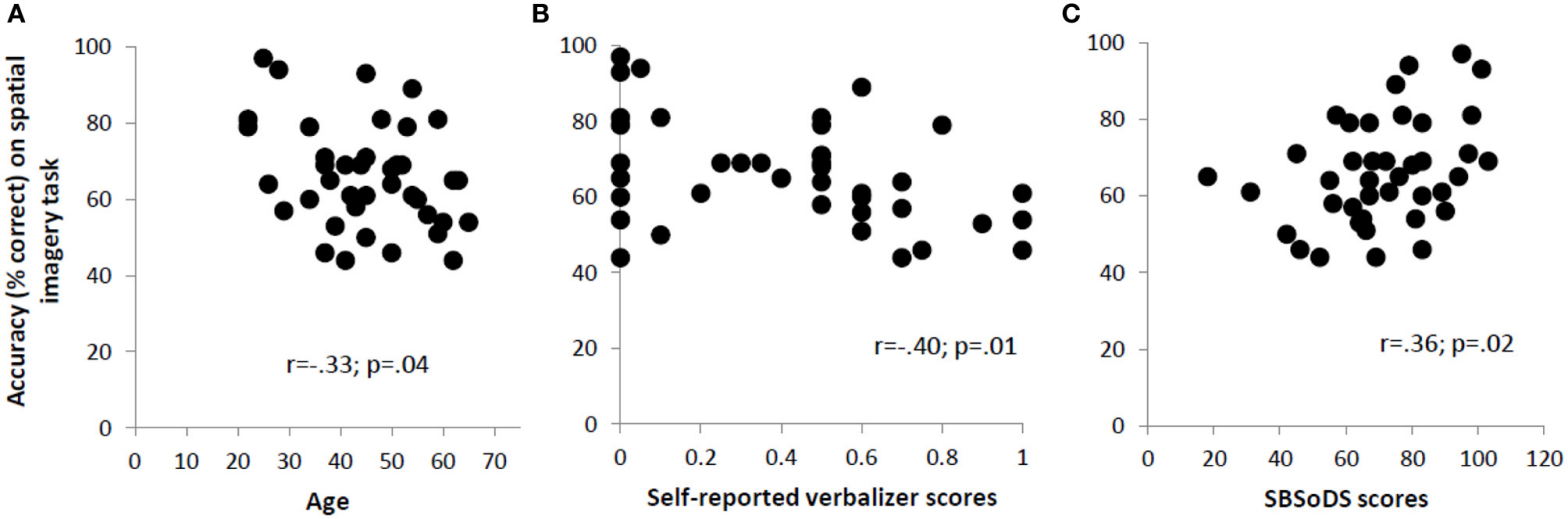
**Significant correlations across all participants between accuracy on the spatial imagery task and (A) age, (B) self-reported verbalizer scores, and (C) SBSoDS scores**.

Since our blind participants varied quite widely in the age at which they lost vision, we examined the correlation between the age at which form vision was completely lost and accuracy on the spatial imagery task for the blind group. This correlation was not significant (*r* = 0.12, *p* = 0.61). However, the age of the participants, regardless of their visual status, affected their performance on the spatial imagery task as indicated by a significant negative correlation (*r* = −0.33, *p* = 0.04), reflecting that accuracy decreased with age (Figure 3A).

The mean self-reported scores for object and spatial imagery did not differ significantly between blind and sighted participants [object imagery: 0.36 vs. 0.44; *t*_(38)_ = −0.87, *p* = 0.39; spatial imagery: 0.13 vs. 0.24; *t*_(38)_ = −1.34, *p* = 0.19]. However, the verbalizer score was higher in blind compared to sighted participants, with the difference trending toward significance [0.51 vs. 0.32; *t*_(38)_ = 1.95, *p* = 0.06]. Next, the correlation between O-S scores (based on self-report, see Materials and Methods) and accuracy on the spatial imagery task was computed: this correlation was not significant for the entire participant group (*r* = −0.23, *p* = 0.16). The correlations were not significant even when individual groups were considered (*r* = −0.18, *p* = 0.44 for the blind and *r* = −0.37, *p* = 0.10 for the sighted), although the data for the sighted suggest a trend toward significance. The self-reported verbalizer scores were significantly negatively correlated with accuracy on the spatial imagery task (*r* = −0.40, *p* = 0.01) (Figure 3B). In order to check whether this correlation could have been driven by the (nearly significant) discrepancy in the verbalizer scores reported by the two groups, a partial correlation, controlling for visual status, was performed. The correlation turned out to still be significant (*r* = −0.34, *p* = 0.03). From analysis of the OSIVQ scores collected on the sighted participants, we observed significant negative correlations between accuracy on the spatial imagery task and, respectively, O-S scores (*r* = −0.59, *p* = 0.006), and verbalizer scores (*r* = −0.75, *p* < 0.001). Age did not correlate with any of the self-reported or OSIVQ scores (*p* > 0.21 for all these correlations).

The SBSoDS scores (69% for the blind vs. 73% for the sighted) did not significantly differ between the two groups [*t*_(38)_ = −0.73, *p* = 0.47]. The correlation between SBSoDS scores and accuracy on the spatial imagery task was significantly positive, across the entire participant group, including both blind and sighted (*r* = 0.36, *p* = 0.02) (Figure 3C). This correlation remained significant even after controlling for the visual status of the participants (partial correlation *r* = 0.37, *p* = 0.02).

## Discussion

In the present study, we showed that the visual status of participants has an impact on performance of the spatial imagery task tested here. The blind were less accurate than the sighted, with no effect of the duration of blindness. These findings suggest that visual loss, even after some visual experience and regardless of its duration, is detrimental to spatial imagery, in keeping with some previous evidence (Vecchi et al., 2004; Noordzij et al., 2007; Cornoldi et al., 2009; Gandhi et al., 2014). In one study, Vecchi et al. (2004) found that sighted and congenitally blind participants performed similarly when patterns in two haptically learned matrices had to be recalled in an integrated whole, but the performance of the blind dropped when they were requested to recall the patterns in two separate matrices. These findings were interpreted to mean that basic spatial processes are intact in the blind, but limitations emerge when the simultaneous maintenance of multiple images is required. This may account for the difference in the present study between blind and sighted participants, since the capability to maintain and update spatial information was crucial in order to efficiently perform the task.

It is worth noting that in the present study, additional processes were required for the spatial imagery task, including the generation of mental images on the basis of the auditory letter cues. The ability to generate mental images has been found to be specifically impaired in aging (Klencklen et al., 2012), possibly due to a decreased selectivity in the activation of category-selective visual areas during imagery (Kalkstein et al., 2011). The difficulty of generating mental images impairs spatial working memory in the elderly (Iachini et al., 2005; Cornoldi et al., 2007). The present finding of a significant negative correlation between age and spatial imagery performance thus seems consistent with previous evidence, and suggests that, besides visual status, age could be an additional factor affecting the performance in a small-scale spatial imagery task, such as the one used here. However, we were able to rule out the possibility that age might have obscured the effects of other individual differences, such as visual status or cognitive style, on spatial imagery performance: blind and sighted participants were not only age-matched, but the correlations computed between age and cognitive style scores for both groups turned out to be insignificant.

The present study also sought to assess whether individual cognitive style affects performance on a spatial imagery task. The data showed a similar distribution of preferences for object and spatial imagery across the different groups, indicating that the distribution of imagery preferences is not affected by visual status. It is worth noting, however, that a marginally significant difference was observed for the self-reported verbalizing scores, with the blind tending to have higher scores than the sighted. Since preferences for object/spatial imagery lie along a continuum rather than being mutually exclusive, we examined correlations of the O-S self-reported scores with performance on the spatial imagery task. However, no overall correlation was found between these two variables. The sighted group did show a trend toward a correlation in the expected direction, with better performance tending to correlate with a higher preference for spatial (vs. object) imagery. This correlation was clearly significant when considering the analysis conducted on the OSIVQ scores, indicating further that our spatial imagery task is a valid index of overall spatial ability. There was no such correlation in the blind, suggesting that although individual preferences exist, vision loss may override their influence on spatial imagery performance. However, this inference should be regarded as tentative in the absence of an OSIVQ-like questionnaire that is suitable for blind people.

The verbalizer scores, whether self-reported (by all participants) or collected with the OSIVQ (in the sighted), were robustly negatively correlated with accuracy on the spatial imagery task. The possibility that this outcome (in the case of the self-reported scores) could simply reflect visual status was ruled out by partial correlation analysis in which this variable was controlled. This finding fits with previous evidence, showing that poor navigators tend to use strategies relying on verbal information and have difficulty in using more efficient allocentric visual map formats (Kato and Takeuchi, 2003; Baldwin and Reagan, 2009; Pazzaglia and Moè, 2013). Very recently, Schmidt et al. (2013) demonstrated that the poorer performance of blind people at remembering information about spatial environments was likely related to their preference for a verbal rehearsal strategy. Even more interestingly, the authors found that this strategy was preferred by blind people with more limited mobility skills. By contrast, blind people who were more independent tended to prefer a mental imagery strategy, just as sighted people did, and performed at the same level as the sighted.

Furthermore, we found a significant positive correlation between the SBSoDS scores and the level of spatial imagery performance. This outcome is of interest for multiple reasons: it provides evidence that the spatial imagery task tested here is ecologically valid and also reinforces the idea that a better sense of direction is related to a more proficient representation of spatial information. It has recently been demonstrated that blind people who are highly independent navigators (e.g., long cane and public transportation users) report high SBSoDS scores, whereas those who are less independent navigators (e.g., relying on a personal driver) report low SBSoDS scores, and individuals who are guide dog users have middle range scores (Halko et al., in press). In their study, Halko and co-workers also showed a positive relationship between SBSoDS scores and activation within the right temporo-parietal junction of early-blind participants while navigating in an indoor virtual environment. Intriguingly, this area is thought to be involved in egocentric-based navigation tasks (Ciaramelli et al., 2010). The present findings suggest that a better sense of direction is related not only to more efficient navigation, but also correlates with a more proficient mental representation of small-scale spatial information (Vandenberg et al., 1985; Wolbers and Hegarty, 2010).

Overall, the present data point to the impact of individual differences on spatial abilities (Bryant, 1982; Miyake et al., 2001; Ozel et al., 2004; Kozhevnikov et al., 2007; Turano et al., 2009). In particular, they suggest that the lower proficiency of late-blind people at processing spatial information could be mediated by factors other than visual deprivation *per se*, such as the use of verbal strategies and/or to poorer mobility skills. Further work should address whether the absence of any visual experience, as in those who are congenitally blind, could give rise to a different pattern of spatial imagery skills. Very recent data suggest that congenitally blind children dramatically improved their performance in a small-scale spatial imagery task after their sight had been restored (Gandhi et al., 2014). Thus, although visual experience is crucial for the adequate development of spatial imagery capabilities, vision retains a good degree of plasticity even after adolescence (Gandhi et al., 2014). A more accurate assessment of what cognitive style preferences are associated with blindness would possibly shed more light on the mechanisms underpinning spatial imagery processes associated with visual deprivation. In this regard, the development of a standardized instrument similar to the OSIVQ that can be used by the blind and visually impaired could be of crucial importance. Moreover, it seems relevant to explore whether rehabilitative approaches for blind people should be tailored to these individual preferences and skills in order to implement more effective strategies for dealing with the environment (Kuyk et al., 2004). For instance, rehabilitation programs including orientation and mobility training and favoring coding of the environment based on its spatial features instead of verbal descriptions might foster more efficient spatial representations in those with inherent preferences for spatial imagery, whereas individuals more inclined to object imagery or verbal coding strategies might benefit from alternative approaches.

## Author contributions

The work was conceived and designed by Simon Lacey and K. Sathian; the data were obtained by Jonathan B. Lin and analyzed by Valeria Occelli and Jonathan B. Lin. Valeria Occelli drafted the manuscript; Valeria Occelli, Simon Lacey, and K. Sathian actively participated in writing and revising the manuscript for publication.

### Conflict of interest statement

The authors declare that the research was conducted in the absence of any commercial or financial relationships that could be construed as a potential conflict of interest.
