# Corrigendum: Loss of form vision impairs spatial imagery

**DOI:** 10.3389/fnhum.2014.00579

**Published:** 2014-08-21

**Authors:** Valeria Occelli, Jonathan B. Lin, Simon Lacey, K. Sathian

**Affiliations:** ^1^Neurology, Emory UniversityAtlanta, GA, USA; ^2^Rehabilitation Medicine, Emory UniversityAtlanta, GA, USA; ^3^Psychology, Emory UniversityAtlanta, GA, USA; ^4^Rehabilitation R&D Center of Excellence, Atlanta VAMCDecatur, GA, USA

**Keywords:** blindness, imagery, sense of direction, cognitive style, spatial

In the Results section, the mean SBSOD scores are erroneously reported as percentages (73 and 69%). The actual values are 73 and 69.

## Conflict of interest statement

The authors declare that the research was conducted in the absence of any commercial or financial relationships that could be construed as a potential conflict of interest.

