# Supplementary material for: Loss of form vision impairs spatial imagery
Source: Front Hum Neurosci. 2014 Mar 19;8:159. doi: 10.3389/fnhum.2014.00159 (PMC3958697; doi:10.3389/fnhum.2014.00159)
Supplement: Supplementary file 1 [file Presentation1.PDF]

Appendix

Thank you for taking part in the research of the Sathian lab. We have one follow-up question – please read the following short explanation and then answer the question.

Research has shown that when people use imagery – imagining something in their mind – they usually prefer one of two kinds of imagery, either object imagery or spatial imagery.

'Object imagers' tend to have detailed images of objects and scenes that tend to concentrate on the literal appearance of objects, including color, texture, patterns, brightness, luminosity, etc., as well as shapes. By contrast, 'spatial imagers' tend to have more schematic images that concentrate on the shape of objects, and how these are transformed. An easy way to think of this difference is the contrast between a photograph (object imagery) and a blueprint or diagram (spatial imagery).

Other people tend not to use imagery and instead rely on language, for example, verbal descriptions of reasoning, in a more analytic style. These people would be described as verbalizers rather than imagers. Here, the difference would be between following written/verbal instructions and following a map/diagram.

With this in mind, which kind of imagery do you think that you typically prefer:

Are you an object imager, a spatial imager, a verbalizer, or a combination of these different types?

If object and spatial imagery were the only options, which would describe you best?
